# A teaching protocol demonstrating the use of EasyClone and CRISPR/Cas9 for metabolic engineering of *Saccharomyces cerevisiae* and *Yarrowia lipolytica*

**DOI:** 10.1093/femsyr/foz062

**Published:** 2019-09-26

**Authors:** N Milne, L R R Tramontin, I Borodina

**Affiliations:** The Novo Nordisk Foundation Center for Biosustainability, Technical University of Denmark, 2800 Kgs. Lyngby, Denmark

**Keywords:** CRISPR/Cas9, EasyClone, metabolic engineering, *Saccharomyces cerevisiae*, *Yarrowia lipolytica*, β-carotene

## Abstract

We present a teaching protocol suitable for demonstrating the use of EasyClone and CRISPR/Cas9 for metabolic engineering of industrially relevant yeasts *Saccharomyces cerevisiae* and *Yarrowia lipolytica*, using β-carotene production as a case study. The protocol details all steps required to generate DNA parts, transform and genotype yeast, and perform a phenotypic screen to determine β-carotene production. The protocol is intended to be used as an instruction manual for a two-week practical course aimed at M.Sc. and Ph.D. students. The protocol details all necessary steps for students to engineer yeast to produce β-carotene and serves as a practical introduction to the principles of metabolic engineering including the concepts of boosting native precursor supply and alleviating rate-limiting steps. It also highlights key differences in the metabolism and heterologous production capacity of two industrially relevant yeast species. The protocol is divided into daily experiments covering a two-week period and provides detailed instructions for every step meaning this protocol can be used ‘as is’ for a teaching course or as a case study for how yeast can be engineered to produce value-added molecules.

## Teaser

A teaching protocol demonstrating how to engineer yeast to produce β-carotene using CRISPR/Cas9.

## INTRODUCTION

Metabolic engineering is a rapidly emerging field, which draws inspiration from multiple engineering disciplines to design and alter metabolic pathways for useful purposes (Stephanopoulos, Aristidou and Nielsen 1998). The budding yeast *Saccharomyces cerevisiae* (*S. cerevisiae*) is frequently used as a host organism for the engineering of metabolic pathways, particularly for the production of value-added molecules, with a large number of reports describing the engineering of this organism for an incredibly diverse range of molecules (Borodina and Nielsen 2014). *Yarrowia lipolytica* (*Y. lipolytica*) is an oleaginous yeast increasingly used for the production of biofuels and chemicals (Darvishi *et al*. 2018). *Y. lipolytica* is especially well suited for industrial production of oleochemicals due to its ability to accumulate lipids up to 70% of dry cell weight as well as high flux through tricarboxylic acid cycle intermediates and cellular precursors such as acetyl-CoA and malonyl-CoA (Beopoulos and Nicaud 2012; Marella *et al*. 2018; Markham and Alper 2018).

A good example of a value-added molecule that was investigated in this protocol is the vitamin A precursor β-carotene. β-Carotene is increasingly used in food and feed additives, cosmetics and health supplements (Li *et al*. 2013), but is predominantly derived from chemical synthesis (Lange and Steinbüchel 2011) using petrochemically derived substrates (Ribeiro, Barreto and Coelho 2011). Biotechnological production of β-carotene via yeast fermentation has the potential to deliver a low-cost, environmentally friendly alternative to chemical synthesis. To this end, there has been a multitude of reports of the engineering of various microorganisms to enable β-carotene production (Rodríguez-Sáiz *et al*. 2007; Verwaal *et al*. 2007; Lange and Steinbüchel 2011; Do Quynh Nguyen *et al*. 2012; Li *et al*. 2013; Yang and Guo 2014; Ronda *et al*. 2015).

Here, we describe a detailed protocol outlining all steps required to introduce β-carotene production in both *S. cerevisiae* and *Y. lipolytica*. The protocol details assembling DNA into integration expression cassettes using the EasyClone method (Jessop-Fabre *et al*. 2016; Holkenbrink *et al*. 2017), and uses the red phenotype from *ADE2* knockout (Ugolini and Bruschi 1996) to demonstrate how to generate and assemble guide RNA vectors for CRISPR-Cas9 genome targeting (Jakočiūnas *et al*. 2015). All necessary plasmids have been deposited at Addgene and all yeast strains have been deposited at Euroscarf.

**Figure 1.**
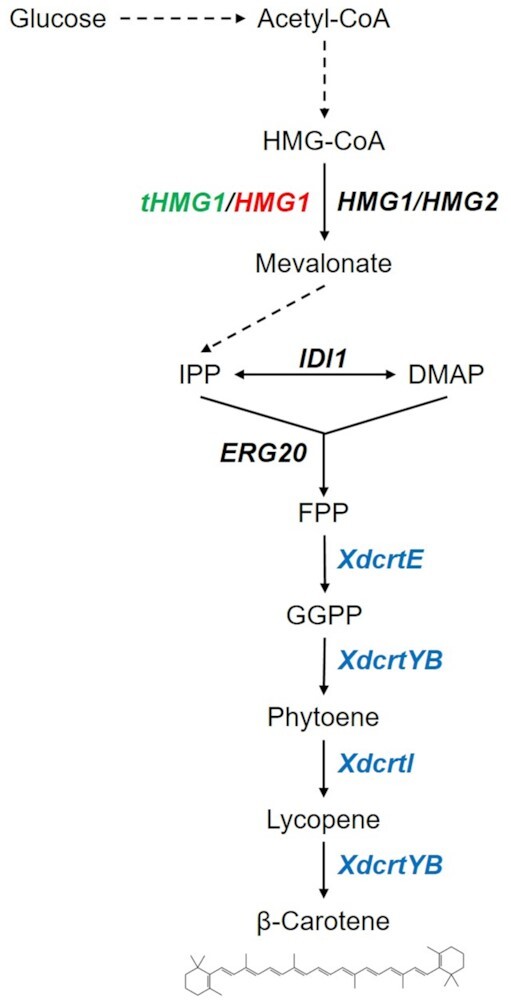
Overview of engineering β-carotene production in *S. cerevisiae* and *Y. lipolytica*. Dashed lines represent multiple grouped reactions. Black: native yeast genes, green: overexpression of truncated native *S. cerevisiae gene* (tHMG1 is a truncated variant of HMG1 with removed feedback inhibition) (Verwaal *et al*. 2007), red: expression of native *Y. lipolytica* gene, blue: expression of heterologous genes from *X. dendrorhous*. HMG-CoA: 3-hydroxy-3-methyl-glutaryl-CoA, IPP: Δ3-isopentenyl-pyrophosphate, DMAP: dimethylallyl-pyrophosphate, trans, trans-farnesyl diphosphate, FPP: farnesyl pyrophosphate, GGPP: geranylgeranyl diphosphate.

β-carotene biosynthesis will be introduced into yeast following the schematic outline in Fig. 1. Via the ergosterol pathway, β-carotene precursor molecule *trans, trans*-farnesyl diphosphate (FPP) is produced using native yeast metabolism. Heterologous genes sourced from *Xanthophyllomyces dendrorhous* (*X. dendrorhous*) then catalyze the conversion of FPP to β-carotene. Expression of heterologous genes *XdcrtE*, *XdcrtYB* and *XdcrtI* results in *de novo* β-carotene production from glucose. To introduce the concept of boosting precursor supply, the protocol describes modifications to increase mevalonate formation by overexpressing a truncated version of HMG1 (tHMG1) in *S. cerevisiae* (previously shown to increase flux through the pathway (Verwaal *et al*. 2007)), and a nontruncated version of HMG1 in *Y. lipolytica* (since the truncated variant was shown to be less efficient (Kildegaard *et al*. 2017)). To introduce the concept of alleviating rate-limiting steps in the metabolic pathway, the protocol also describes the introducing of an additional copy of a known rate-limiting step in β-carotene biosynthesis (*XdcrtI*) (Verwaal *et al*. 2007).

From a genome editing perspective, one key advantage of *S. cerevisiae* is its inherent ability to reliably integrate heterologous pieces of DNA through homologous recombination. This is especially useful when combined with the CRISPR-Cas9 system, which promotes the homologous recombination by introducing double-strand DNA breaks. Double-strand DNA breaks also serve as a selection, where only the cells that repair the break can survive. This eliminates the need to use selection markers (Norville *et al*. 2013). This is demonstrated in the protocol by the simultaneous integration of expression cassettes at up to three separate loci in the *S. cerevisiae* genome, allowing us to take a wild-type *S. cerevisiae* strain and convert it into a β-carotene producer in a single transformation step.

**Figure 2.**
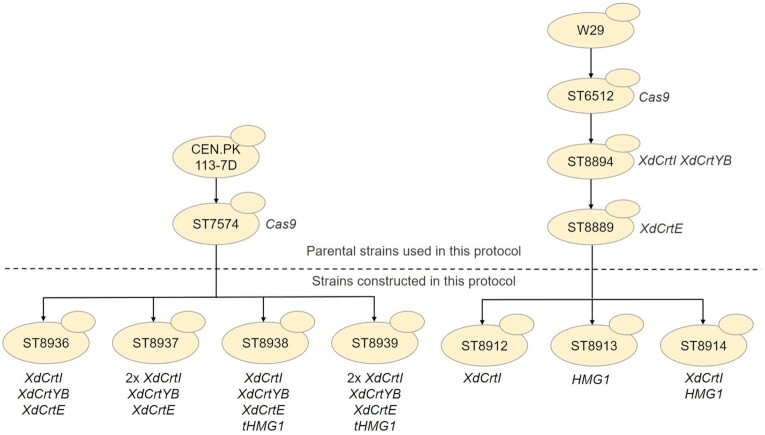
Strain genealogy diagram showing the strain construction procedure in *S. cerevisiae* (left) and *Y. lipolytica* (right).

While new metabolic engineering tools to facilitate genetic modifications in *Y. lipolytica* have emerged in the past years, it is still challenging to obtain multiple genome edits in a single transformation event with good efficiency (Holkenbrink *et al*. 2017). This can be due to the lower transformation efficiency of this yeast and/or due to lower efficiency of homologous recombination. Therefore, the engineering strategy used for *Y. lipolytica* in this protocol instead relies on single genome integration of expression cassettes. The parental *Y. lipolytica* strain used in this protocol (ST8889) already has the β-carotene pathway integrated in the genome and thus already produces β-carotene, and additionally, contains a *KU70* deletion (*ku70∆*) to enhance the frequency of homologous recombination by disrupting the Ku70-Ku80 homodimer complex responsible for catalyzing nonhomologous end joining (Kretzschmar *et al*. 2013). To demonstrate the concept of boosting precursor supply and overcoming a rate-limiting step, three plasmids containing the genes YlHMG1 and *XdcrtI* are constructed and integrated separately in the parent strain. An overview of the strain construction genealogy for both *S. cerevisiae* and *Y. lipolytica* is shown in Fig. 2.

This protocol was used as a teaching material for the Advanced Experimental Synthetic Biology for Cell Factories course at the Novo Nordisk Foundation Center for Biosustainability at the Technical University of Denmark.

## MATERIALS

### *S. cerevisiae*, *Y. lipolytica* and *Escherichia coli* strains

Parental *S. cerevisiae* and *Y. lipolytica* strains required for this protocol are available from Euroscarf. A full list of strains (including those constructed in this protocol), and their respective Euroscarf identifiers can be found in the supplementary materials (Supporting Information).

- Chemically competent *DH5α E. coli* cells for plasmid transformation and propagation
- Prototrophic strain of *S. cerevisiae* strain containing *cas9* (ST7574) derived from CEN.PK113–7D (Entian and Kötter 2007)
- Prototrophic strain of *Y. lipolytica* containing *cas9*, *ku70Δ* with a single copy of the heterologous β-carotene pathway (ST8889) derived from W29 strain (ATCC20460) (Pomraning and Baker 2015)

### Plasmids

All plasmids required for this protocol can be obtained from Addgene. A full list of plasmids (including those constructed in this protocol), and their respective Addgene identifiers can be found in the supplementary materials (Supporting Information).

### Equipment

- Thermocycler
- Gel electrophoresis equipment for DNA separation
- Safe Imager 2.0 Blue-Light Transilluminator (ThermoFisher Scientific, MA, USA)
- NanoDrop (ThermoFisher Scientific, MA, USA) for DNA quantification
- 30, 37 and 60°C standing incubators
- 30 and 37°C shaking incubators
- Heating blocks with a range up to 100°C
- Blue light or UV transilluminator
- Spectrophotometer for optical density measurements (Implen Nanophotometer, (Implen, Germany) or similar)
- Centrifuge (Thermo Heraeus multifuge XI (ThermoFisher Scientific, MA, USA) or similar) with 50 mL Falcon tube rotor capable of up to 4000 g
- Microcentrifuge
- HPLC machine (ThermoFisher Scientific, MA, USA or similar) with a Discovery HS F5 150 mm x 2.1 mm column (particle size 3 mm)
- 24-deep-well plates (ThermoFisher Scientific, MA, USA)
- Air penetrable sandwich cover for 24-deep-well plates (Enzyscreen B.V. The Netherlands)
- 250 mL shake flasks (as an alternative to 24-deep-well plates)
- 10 mL preculture tubes (Greiner Bio, Austria)
- 96-deep-well plate (ThermoFisher scientific, MA, USA)
- Air penetrable sandwich cover for 96-deep-well plates (Enzyscreen B.V. The Netherlands)
- PCR tubes (ThermoFisher Scientific, MA, USA; 11667009001)
- Gene Ruler 1 Kb DNA ladder (Sigma Aldrich, MO, USA; D0428)
- Precellys R 24 homogenizer (Bertin Corp, MD, USA)
- Rotatory evaporator (SpeedVac)

### Chemicals

- Bacto yeast extract (Difco-ThermoFisher Scientific, MA, USA; 212750)
- Bacto agar (Difco-ThermoFisher Scientific, MA, USA; 214010)
- Bacto peptone (Difco-ThermoFisher Scientific, MA, USA; 211677)
- Yeast Nitrogen Base without amino acids (Sigma Aldrich, MO, USA; Y0626)
- Yeast Synthetic Drop-out Medium Supplements w/o uracil (Sigma Aldrich, MO, USA; Y1501)
- D-Glucose (Sigma Aldrich, MO, USA; G7021)
- Ammonium sulfate (VWR, Denmark; 21 333.296)
- Potassium phosphate monobasic (Sigma Aldrich, MO, USA; 60220)
- Magnesium sulfate heptahydrate (VWR, Denmark; A14491.0B)
- Lithium acetate dehydrate (Sigma Aldrich, MO, USA; L6883)
- Sodium dodecyl sulfate (Sigma Aldrich, MO, USA; L4390)
- Polyethylene glycol 3350 (Sigma Aldrich, MO, USA; P4338)
- Absolute ethanol (Sigma Aldrich, MO, USA; 493 511)
- Deoxyribonucleic acid from salmon testes (Sigma Aldrich, MO, USA; D9156)
- Ethyl acetate (Sigma Aldrich, MO, USA; 270989)
- 3,5-di-tert-4-butylhydroxytoluene (BHT) (Sigmas Aldrich, MO, USA; 47168)
- Ammonium formate (Sigma Aldrich, MO, USA; 516961)
- Formic acid (Sigma Aldrich, MO, USA; F0507)
- Acetonitrile (Sigma Aldrich, MO, USA; 271004)
- β-carotene (Sigma Aldrich, MO, USA; C4582)
- Single-stranded DNA (ssDNA) (Sigma Aldrich, MO, USA; D9156)

### Molecular biology reagents and consumables

- DNA purification kits
  - NucleoSpin® Gel and PCR Clean-up kit (Macherey-Nagel, Germany; 740609.250)
  - NucleoSpin Plasmid kit (Macherey-Nagel, Germany; 740588.250)
  - Quick DNA Fungal/bacterial miniprep kit (Zymo Research, CA, USA; D6005)
- PCR reagents
  - PhusionU high-fidelity DNA polymerase (ThermoFisher Scientific, MA, USA; F-555S)
  - 5x Phusion HF buffer (ThermoFisher Scientific, MA, USA; F-518 L)
  - OneTaq® Quick-Load® 2X Master Mix with Standard Buffer (New England Biolabs, MA, USA; M0486L)
  - dNTPs (10 mM) (ThermoFisher Scientific, MA, USA; R0181)
  - MilliQ or RNase free water
- Primers

A full list of primers used in this study can be found in the supplementary materials (Supporting Information).

- Restriction enzymes
  - FastDigest DpnI (ThermoFisher scientific, MA, USA; FD1704)
  - FastDigest NotI (ThermoFisher scientific, MA, USA; FD0594)
  - FastDigest SfaAI (AsiSI) (ThermoFisher scientific, MA, USA; FD2094)
  - Nb.BsmI (New England Biolabs, MA, USA; R0706S)
- GeneRuler 1 Kb DNA Ladder, ready-to-use (ThermoFisher scientific, MA, USA; SM0313)
- 50x TAE Buffer (ThermoFisher scientific, MA, USA; B49)
- TopVision Agarose (ThermoFisher scientific, MA, USA; R0492)
- USER® Enzyme (New England Biolabs, MA, USA; M5505L)
- 10x CutSmart® Buffer (New England Biolabs, MA, USA; B7204S)
- NEBuffer 3.1 (New England Biolabs, MA, USA; B7203S)
- RedSafe Nucleic acid staining solution 20 000x (Intron biotechnology, South Korea)
- 6x DNA loading dye (Life technologies, CA, USA; R0611)
- 2 mL microtube (Sarstedt, Germany)
- 0.5–0.75 mm glass beads (Sigma Aldrich, MO, USA; G1277)

### Liquid growth medium

- LB medium: For preparation of 1 L LB medium, mix 10 g Bacto tryptone, 5 g Bacto yeast extract and 10 g sodium chloride and fill up to 1 L with demineralized water. For solid medium add 2% (w/v) Bacto agar. Heat sterilize for 20 min at 121°C. After sterilization add antibiotics as required.
- YPD (yeast peptone dextrose) medium: For 1 L YP medium, mix 5 g Bacto yeast extract, 10 g Bacto peptone and fill up to 1 L with demineralized water. For solid medium, add 2% (w/v) Bacto agar. Heat sterilize for 20 min at 121°C. After sterilization, add sterilized glucose solution to a final concentration of 2% (w/v) (YPD) and add antibiotics as required.
- SM (synthetic medium): For 1 L of synthetic medium, start with 750 mL of demineralized water and add 5 g ammonium sulfate [(NH_4_)_2_SO4], 3 g monopotassium phosphate [KH_2_PO_4_] and 0.5 g magnesium sulfate heptahydrate [MgSO_4_.7·H_2_O] and add trace elements according to Verduyn *et al*. (1992) and as described below. Dissolve salts and set the pH to 6.0 with 2 M potassium hydroxide [KOH], add demineralized water to reach a final volume of 1 L and heat sterilize for 20 min at 121°C. After sterilization, add vitamins according to Verduyn *et al*. (1992) and as described below.
- Trace metal solution for synthetic media: for 1 L of trace metal solution.

**Table:** 

Chemical	Amount (g)
CaCl_2_.2H2O (Sigma Aldrich, MO, USA; C8106)	4.5
ZnSO_4_.7H2O (Sigma Aldrich, MO, USA; 31665)	4.5
FeSO_4_.7H2O (Sigma Aldrich, MO, USA; 31236)	3
H_3_BO_3_ (Sigma Aldrich, MO, USA; 31146)	1
MnCl_2_.4H2O (Sigma Aldrich, MO, USA; M8054)	1
Na_2_MoO_4_.2H2O (Sigma Aldrich, MO, USA; M1651)	0.4
CoCl_2_.6H2O (Sigma Aldrich, MO, USA; 31277)	0.3
CuSO_4_.5H2O (Sigma Aldrich, MO, USA; C8027)	0.1
KI (Sigma Aldrich, MO, USA; 30315)	0.1
EDTA (Sigma Aldrich, MO, USA; E6758)	15

Dissolve all chemicals listed above except for EDTA one-by-one while maintaining the pH at 6.0 in 900 mL H_2_O. Add the EDTA and gently heat the solution until completely dissolved. Adjust the final pH to 4.0 and the volume to 1 L. Heat sterilize for 20 min at 121°C and store at 4°C. Add 2 mL per 1 L of synthetic medium.

- Vitamin solution for synthetic media: for 1 L of vitamin solution.

Dissolve biotin in 20 mL 0.1 M NaOH and then add 900 mL water. Adjust pH to 6.5 with HCl and add the remaining vitamins. Readjust the pH to 6.5 just before and after adding myo-inositol. Adjust to a final volume of 1 L. Filter sterilize and store at 4°C. Add 1 mL of vitamin solution per 1 L of synthetic media.

**Table:** 

Chemical	Amount
Biotin (Sigma Aldrich, MO, USA; B4639)	50 mg
4-aminobenzoic acid (Sigma Aldrich, MO, USA; 100536)	200 mg
Nicotinic acid (Sigma Aldrich, MO, USA; 72309)	1 g
Ca-Pantotenate (Sigma Aldrich, MO, USA; C8731)	1 g
Pyridoxine HCl (Sigma Aldrich, MO, USA; P6280)	1 g
Thiamine HCl (Sigma Aldrich, MO, USA; T1270)	1 g
myo-Inositol (Sigma Aldrich, MO, USA; I7508)	25 g

### Solid growth medium

Prepared as above for liquid growth medium but with the addition of 20 g L^−1^ agar

### Supplements for selection

- 100 mg L^−1^ Ampicillin (Sigma Aldrich, MO, USA; A9518)
- 100 mg L^−1^ Nourseothricin (Jena Bioscience, Germany; AB-101)
- 200 mg L^−1^ G418 (Sigma Aldrich, MO, USA; G8168)

## PROTOCOL

### Molecular biology preparation before starting the course

We recommend preparing the following before commencing with the experimental protocol:

1. Isolate the plasmids listed in Table 1. Inoculate 5 mL LB + Ampicillin with *E. coli* strains harboring each plasmid in a sterile 10 mL culture tube, incubate overnight at 37°C with shaking at 200 RPM and then purify the plasmid DNA using NucleoSpin Plasmid kit (or similar) according to the manufacturer's instructions.
2. Isolate *S. cerevisiae* and *Y. lipolytica* genomic DNA for use as PCR template. Inoculate 5 mL YPD with parental strains ST7574 and ST8889, incubate overnight at 30°C with shaking at 200 RPM and then purify the genomic DNA using Quick DNA Fungal/bacterial miniprep kit (or similar) according to the manufacturer's instructions. Approximately, 150 ng of *S. cerevisiae* and 100 ng of *Y. lipolytica* DNA are required per person.
3. Prepare ‘USER ready’ EasyClone-MarkerFree backbone plasmids according to Jensen *et al*. (2014) and Holkenbrink *et al*.(2017) and as described below. Approximately, 200 ng of each backbone is needed per person.
  - Digest each EasyClone backbone vector (pCfB3035, pCfB2909, pCfB2904, pCfB6684 and pTAJAK-71) with SfaAI at 37°C for 1 h as follows:
    - 20 μg plasmid DNA
    - 16 U Fast Digest SfaAI (typically 16 μL)
    - 20 μL 10x FastDigest buffer
    - H_2_O to a final volume of 200 μL
  - Mix digested plasmid with DNA loading dye and load the whole product on a 1% agarose 1x TAE gel with 1x RedSafe nucleic acid staining solution, include a GeneRuler 1 Kb DNA ladder. Run at 110 V (300 mA) for 30 min. Excise the digested plasmid using a Safe Imager 2.0 Blue-Light Transilluminator and purify using a NucleoSpin Gel and PCR Clean-up kit (or similar) according to the manufacturer's instructions. Measure the concentration using NanoDrop (or similar).
  - Digest the whole purified digested plasmid with Nb.BsmI at 65°C for 1 h as follows:
    - 1 U Nb.BsmI per μg of digested vector (typically 1 μL/μg)
    - Appropriate volume of NEB buffer 3.1
  - Gel purify as per previous step.
4. Boil single stranded DNA (ssDNA) for 10 min and then store at −20°C until needed for transformation. Approximately, 300 μL per person is needed.
5. Prepare 1 M and 2 M LiAc solutions and 50% (w/v) PEG 3500. Approximately, 400 μL, 50 μL and 3 mL, respectively, per person is required.
6. Prepare solid and liquid cultivation media using the recipes given above. An estimation of the amount required per person is given in brackets.
  - Liquid media
    - LB + Ampicillin (100 mL)
    - YPD (2% glucose) (20 mL)
    - YPD (8% glucose) (40 mL)
    - YPD + G418 (55 mL)
  - Solid media
    - LB + Ampicillin (14 plates/350 mL)
    - YPD (1 plate/25 mL)
    - YPD + G418 (1 plate/25 mL)
    - YPD + G418 + Nourseothricin (10 plates/250 mL)
    - YPD + Nourseothricin (for *Y. lipolytica*) (6 plates/150 mL)
7. Since most steps in this protocol are time demanding, we also strongly recommend preparing backups of each step in the protocol should any experiment fail.

**Table 1.** Plasmids to isolate in advance for use in protocol.

Name	Relevant characteristics
Templates for PCR amplification (∼50 ng per person required)	
p1977	pUC19 + *pTDH3-pTEF1*
pCfB8739	pUC19 + *XdcrtYB*
pCfB8740	pUC19 + *XdcrtI*
pCfB8741	pUC19 + *XdcrtE*
pCfB8742	pUC19 + *pGPD1-pFBA1*
pCfB8818	pUC19 + *YlHMG1* (from *Y. lipolytica*)
pCfB8792	pUC19 + *pSNR52-ADE2* gRNA*-tSUP4*
gRNA plasmids for targeting genomic integration sites (∼2 μg per person required)	
pCfB5191	2 μm ori NatMX *pSNR52*-X-4 gRNA-*tSUP4 pSNR52*-XII-5 gRNA-*tSUP4*
pCfB8622	2 μm ori NatMX *pSNR52-ADE2* gRNA-*tSUP4*
pCfB3052	2 μm ori NatMX *pSNR52*-X-4 gRNA-*tSUP4 pSNR52*-XII-5 gRNA-*tSUP4 pSNR52*-XI-3 gRNA-*tSUP4*
pCfB6631	2 μm ori NatMX *pSNR52*-IntD1 gRNA-*tSUP4*
Episomal yeast expression plasmids (∼1 μg per person required)	
pCfB2312	2 μm ori c*as9* KanMX
pTAJAK-71	2 μm ori NatMX
pCfB3405	2 μm ori NatMX
Backbone plasmids for EasyClone-MarkerFree plasmid assembly	
pCfB3035	pX-4-USER
pCfB2909	pXII-5-USER
pCfB2904	pXI-3-USER
pCfB6684	pIntD_1-USER

### Monday (Week 1, Day 1)

**Figure 3.**
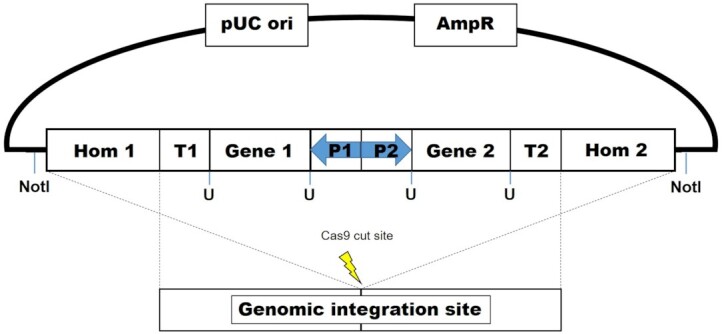
Schematic overview of cloning using the EasyClone system and genomic integration using CRISPR-Cas9 and homologous recombination. Biobricks containing complementary overhangs are fused (at sites designated with a ‘U’) using USER cloning to plasmid backbones containing terminators and homology arms flanked by NotI restriction sites. NotI digested overexpressions are integrated at genomic landing pads by CRISPR-Cas9 mediated repair of double-strand breaks at the genomic integration site.

Today students will PCR amplify DNA biobricks and assemble them into EasyClone-MarkerFree plasmids for transformation into yeast (Jensen *et al*. 2014; Holkenbrink *et al*. 2017). DNA parts to amplify include the heterologous β-carotene biosynthetic genes *XdcrtI*, *XdcrtE* and *XdcrtYB* (Verwaal *et al*. 2007), a truncated version of native *S. cerevisiae* HMG1 gene (tHMG1), reported to remove feedback regulation (Verwaal *et al*. 2007), the native HMG1 gene from *Y. lipolytica* (YlHMG1), and strong constitutive yeast promoters for expression of each gene. While plasmids containing gRNA cassettes for targeting integration fragments into the yeast genome are supplied, to demonstrate how to generate and assemble gRNA expression plasmids, students will also PCR amplify an *ADE2* gRNA expression cassette and assemble it into a gRNA plasmid backbone using the EasyClone system. Different gene combinations (outlined in Table 3) will be constructed to demonstrate how they impact β-carotene production. EasyClone-MarkerFree is a DNA assembly and genome integration method based on Uracil-Specific Excision Reagent (USER) cloning and yeast homologous recombination at predefined genomic landing pads. Briefly, USER cloning is a directional cloning technique where a short predefined (6–10 bp) homology arm starting with a single deoxyuridine (dU) residue is placed at the 5′ end of each primer. After amplification of the DNA fragments to be fused with these primers, the dU residue is cleaved by a USER mix to generate complementary 3′ single stranded overhangs that can be fused together (Nour-Eldin, Geu-Flores and Halkier 2010). The EasyClone system uses standardized overhangs to fuse up to two genes of interest to a specific promoter and terminator in a single plasmid. Furthermore, plasmid backbones contain long (500 bp) homology arms flanked by NotI restriction sites allowing the USER assembled expression cassette to integrate at predefined landing pads in the genome using yeast's own homologous recombination machinery (Jensen *et al*. 2014). These genomic landing pads have been previously validated for growth impairment and strong expression and are placed between essential genetic elements resulting in cell death should loop-out of the inserted fragments occur (Mikkelsen *et al*. 2012; Holkenbrink *et al*. 2017). Marker-free selection is achieved using CRISPR-Cas9 where a targeted double-strand break (DSB) at the genomic landing pads creates a lethal event that can be repaired by integration of the expression cassette (Jessop-Fabre *et al*. 2016). A schematic overview of the cloning and genomic integration procedure is shown in Fig. 3.

1. PCR amplify biobricks for EasyClone and gRNA plasmid assembly listed in Table  2 according to the scheme listed below. Up to 500 ng of each biobrick is required for EasyClone plasmid assembly, so we recommend doing some biobrick amplifications 2x.
  - Set up the following PCR mixture for each biobrick.

**Table:** 

Reagent	Amount
H_2_O	33 μL
5x Phusion HF Buffer	10 μL
Fwd primer (10 μM)	2.5 μL
Rev primer (10 μM)	2.5 μL
dNTP mix (10 mM)	1 μL
Template	50 ng
PhusionU polymerase*	1 U

* The EasyClone plasmid assembly system utilizes USER cloning to join DNA pieces together. It is thus vital to use a polymerase that can recognize the uracil base encoded in the primers.

  b. Run the PCR according to the following conditions.

**Table:** 

	Temp (°C)	Time (s)
	98	60
30x	98	10
	55	30
	72	240
	72	300

  c. After the PCR has finished, mix each of the PCR products with DNA loading dye and load the whole product on a 1% agarose 1x TAE gel with 1x RedSafe nucleic acid staining solution, include a GeneRuler 1 Kb DNA ladder. Run at 110 V (300 mA) for 30 min. A gel image of a successful biobrick PCR is shown in Fig. 4.
  d. Excise the PCR products (after confirming the expected size) using a Safe Imager 2.0 Blue-Light Transilluminator and purify using a NucleoSpin Gel and PCR Clean-up kit (or similar) according to the manufacturer's instructions, elute the DNA in a final volume of 25 μL.
  e. Measure the concentration of purified biobricks using NanoDrop (or similar).
2. Assemble plasmids outlined in Table 3 using the EasyClone method according to Jensen *et al*. (2014) and Holkenbrink *et al*.(2017) and as outlined below. We recommend including controls where only the plasmid backbone is added to account for false positives caused either by undigested plasmid or re-ligation of the plasmid.
  - Prepare reaction mixtures as follows:
    - 1 μL 10 x CutSmart buffer
    - 1 U USER enzyme (typically 1 μL)
    - 100 ng of each Biobrick
    - 50 ng of plasmid backbone
    - Up to 10 μL H_2_O*
      *Scale the final reaction volume up or down as required.
  - Run in a PCR machine according to the following protocol.
  - Transform assembled plasmids into chemically competent *E. coli* DH5α as follows:
    - Thaw chemically competent cells on ice for ∼20 min.
    - Add 50 μL of cells to each reaction.
    - Heat shock at 42°C for 90 s and then cool on ice for 2 min.
    - Plate cells on LB + Ampicillin and incubate at 37°C overnight.

**Table 2.** Biobricks to amplify.

#	Biobrick	Fwd primer	Rev primer	Size	Template
1	BB01567 (*XdcrtYB*)	PR-7039	PR-7040	2039	pCfB8739
2	BB01568 (*XdcrtI*)	PR-7041	PR-7042	1766	pCfB8740
3	BB01569 (*XdcrtE*)	PR-7043	PR-7044	1148	pCfB8741
4	BB3287 (*tHMG1*)	PR-22921	PR-22922	1607	*S. cerevisiae* gDNA
5	BB0464 (←*pTDH3-pTEF1*→)	PR-1853	PR-22409	1146	p1977
6	BB0009 (*pPGK1*→)	PR-22406	PR-22407	984	*S. cerevisiae* gDNA
7	BB0410 (←*pTDH3*)	PR-1852	PR-1853	715	*S. cerevisiae* gDNA
8	BB3787 (*HMG1*)	PR-23752	PR-23753	3000	pCfB8818
10	BB2212 (←*pGPD1-pFBA1*→)	PR-13338	PR-15524	1789	pCfB8742
11	BB1244 (←*pGPD1*)	PR-13337	PR-13338	949	*Y. lipolytica* gDNA
12	BB1559 (*pFBA1*→)	PR-15523	PR-15524	846	*Y. lipolytica* gDNA
13	BB3713 (*ADE2* gRNA knockout cassette)	PR-10525	PR-10529	432	pCfB8792

**Table 3.** Plasmids to assemble using EasyClone.

#	Plasmid name	Backbone	Gene 1	Promoter(s)	Gene 2
1	pCfB8379 (*X-4::XdcrtI-XdcrtYB*)	pCfB3035	BB01568 (*XdcrtI*)	BB0464 (←pTDH3-pTEF1→)	BB01567 (*XdcrtYB*)
2	pCfB8380 (*XII-5::XdcrtE*)	pCfB2909		BB0009 (pPGK1→)	BB01569 (*XdcrtE*)
3	pCfB8381 (*XI-3::XdcrtI-tHMG1*)	pCfB2904	BB01568 (*XdcrtI*)	BB0464 (←pTDH3-pTEF1→)	BB3287 (*tHMG1*)
4	pCfB8382 (*XI-3::XdcrtI*)	pCfB2904	BB01568 (*XdcrtI*)	BB0410 (←pTDH3)	
5	pCfB8383 (*XI-3::tHMG1*)	pCfB2904		BB0009 (pPGK1→)	BB3287 (*tHMG1*)
6	pCfB8786 (*IntD1::XdcrtI)*	pCfB6684	BB01568 (*XdcrtI*)	BB1244 (←pGPD1)	
7	pCfB8787 (*IntD1::HMG1*)	pCfB6684		BB1559 (pFBA1→)	BB3787 (*HMG1*)
8	pCfB8788 (*IntD1::XdcrtI-HMG1)*	pCfB6684	BB01568 (*XdcrtI*)	BB2212 (←pGPD1-pFBA1→)	BB3787 (*HMG1*)
9	pCfB8622 (NatMX_ADE2_gRNA)	pTAJAK-71	BB3713 (ADE2 gRNA knockout cassette)		
10	Backbone control	pCfB3035			
11	Backbone control	pCfB2909			
12	Backbone control	pCfB2904			
13	Backbone control	pCfB6684			
14	Backbone control	pTAJAK-71			

NB. While some protocols recommend a recovery step in LB or SOC media before plating *E. coli* cells on LB + Ampicillin, we do not recommend this as it can increase the occurrence of false positives.

Alternate protocol

1. If the total number of *E. coli* colonies on the transformation plate is low it may be due to poor chemical competency. To increase the transformation efficiency, after heat shock, incubate cells in 500 μL of LB or SOC media before plating.

### Tuesday (Day 2)

Today, students will confirm correct assembly of EasyClone plasmids by colony PCR (cPCR). Students will then set up overnight *E. coli* cultures of clones to further confirm correct assembly by Sanger sequencing.

1. Set up colony cPCR reactions listed in Table 4 to confirm correct assembly of EasyClone plasmids.
  - Set up the following PCR mixture for each clone. We recommend testing three clones from each EasyClone reaction.
    - 10 μL OneTaq Quick-Load 2x Master Mix with Standard Buffer
    - 1 μL of each primer (10 μM)
    - Add H_2_O to a final volume of 20 μL.
  - Transfer a small amount of cells to each PCR reaction using a toothpick (circle and number each colony with a marker pen before picking).

**Table:** 

Temp (°C)	Time (min)
37	25
25	10
20	10
15	10

  c. Run in a PCR machine according to the following protocol.

**Table:** 

	Temp (°C)	Time (s)
	95	300
30x	94	30
	52	30
	68	150
	68	300

  d. After the PCR has finished load 5 μL directly onto a 1% agarose 1x TAE gel with 1x RedSafe nucleic acid staining solution, include a GeneRuler 1 Kb DNA ladder. Run at 110 V (300 mA) for 30 min and analyze fragment size on a UV transilluminator. Note particularly for pCfB8381 that the expected bands are very close together. To achieve clear separation of these bands, longer run times may be required. An example of a successful cPCR confirming correct EasyClone assembly is given in Fig. 5.
  e. Inoculate 5 mL LB + Ampicillin with the correct clones and incubate overnight at 37°C with shaking at 200 RPM. If possible, we recommend inoculating two correct clones per plasmid for further testing.

**Figure 4.**
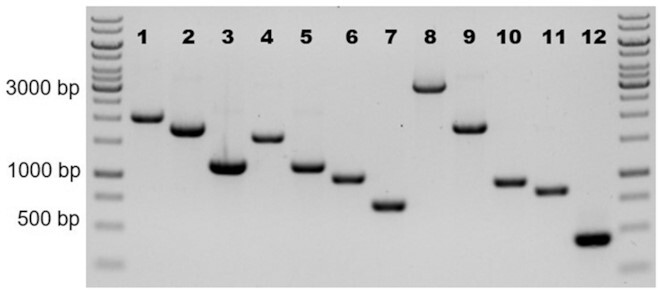
Gel image of a successful biobrick PCR amplification. Numbers correspond to those presented in Table 2. Negative image given for clarity.

**Figure 5.**
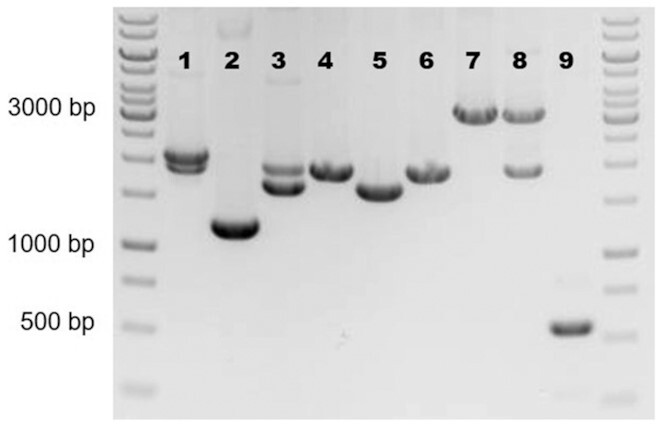
Gel image of a successful colony PCR amplification and correct assembly of each EasyClone plasmid. Numbers correspond to those presented in Table 4. Negative image given for clarity.

**Table 4.** cPCR setup to confirm EasyClone plasmid assembly.

		PCR reaction				
#	Plasmid cPCR	Primer 1	Primer 2	Primer 3	Primer 4	Correct size
1	pCfB8379 (*X-4::XdcrtI-XdcrtYB*)	PR-22955	PR-224	PR-339	PR-225	2467/2087
2	pCfB8380 (*XII-5::XdcrtE*)	PR-340	PR-225			1208
3	pCfB8381 (*XI-3::XdcrtI-tHMG1*)	PR-22955	PR-224	PR-339	PR-225	2467/1655
4	pCfB8382 (*XI-3::XdcrtI*)	PR-22955	PR-224			2467
5	pCfB8383 (*XI-3::tHMG1*)	PR-340	PR-225			1655
6	pCfB8786 (*IntD1::XdcrtI)*	PR-14441	PR-14617			1933
7	pCfB8787 (*IntD1::HMG1*)			PR-15587	PR-14619	3000
8	pCfB8788 (*IntD1::XdcrtI-HMG1)*	PR-14441	PR-14617	PR-15587	PR-14619	1933/3000
9	pCfB8622 (NatMX_ADE2_gRNA)	PR-23875	PR-23876			521

Alternate protocol

1. Using four primers per reaction to generate two PCR products can be difficult, particularly when the band sizes are so close together. It may therefore be beneficial to separate these PCR reactions.
2. If no bands are observed from the cPCR, inoculate 5 mL LB + Ampicillin with several clones from each EasyClone assembly and incubate overnight at 37°C with shaking at 200 RPM. Follow the protocol below to purify and then NotI digest the plasmids. Load 5 μL of NotI digested plasmid onto a 1% agarose 1x TAE gel with 1x RedSafe nucleic acid staining solution, include a GeneRuler 1 Kb DNA ladder. Run at 110 V (300 mA) for 30 min and analyze fragment size on a UV transilluminator. The predicted sizes of correctly assembled NotI digested fragments are given in Table 5; note that there will also be a band at ∼2800 bp corresponding to the plasmid backbone. Also, note that pCfB8622 (NatMX_ADE2_gRNA) is an expression plasmid not an integration plasmid and thus does not contain NotI sites for digestion.

**Table 5.** Predicted sizes of integration fragments after NotI digestion.

Plasmid	Size (bp)
pCfB8379 (*X-4::XdcrtI-XdcrtYB*)	6484
pCfB8380 (*XII-5::XdcrtE*)	3852
pCfB8381 (*XI-3::XdcrtI-tHMG1*)	6182
pCfB8382 (*XI-3::XdcrtI*)	3283
pCfB8383 (*XI-3::tHMG1*)	3212
pCfB8786 (*IntD1::XdcrtI)*	5016
pCfB8787 (*IntD1::HMG1*)	6177
pCfB8788 (*IntD1::XdcrtI-HMG1)*	8887

**Table 6.** Outline for yeast transformation.

#	Introduction	Integration fragments	gRNA vector	Marker	Integration site	Plate	Parental strain
1	Beta-carotene pathway	pCfB8379 (*X-4::XdcrtI-XdcrtYB*)	pCfB5191	NatMX	X-4/XII-5	YPDGN	ST7574
		pCfB8380 (*XII-5::XdcrtE*)					
2	Beta-carotene pathway + Extra *XdcrtI*	pCfB8379 (*X-4::XdcrtI-XdcrtYB*)	pCfB3052	NatMX	X-4/XII-5/XI-3	YPDGN	
		pCfB8380 (*XII-5::XdcrtE*)					
		pCfB8382 (*XI-3::XdcrtI*)					
3	Beta-carotene pathway + tHMG1	pCfB8379 (*X-4::XdcrtI-XdcrtYB*)	pCfB3052	NatMX	X-4/XII-5/XI-3	YPDGN	
		pCfB8380 (*XII-5::XdcrtE*)					
		pCfB8383 (*XI-3::tHMG1*)					
4	Beta-carotene pathway + extra *XdcrtI* + tHMG1	pCfB8379 (*X-4::XdcrtI-XdcrtYB*)	pCfB3052	NatMX	X-4/XII-5/XI-3	YPDGN	
		pCfB8380 (*XII-5::XdcrtE*)					
		pCfB8381 (*XI-3::XdcrtI-tHMG1*)					
5	ADE2 knockout	PR-23 173 (ADE2 dsDNA repair fragment)	pCfB8622	NatMX	ADE2	YPDGN	
6	EasyClone integration minus control		pCfB5191	NatMX	X-4/XII-5	YPDGN	
7	EasyClone integration minus control		pCfB3052	NatMX	X-4/XII-5/XI-3	YPDGN	
8	ADE2 knockout minus control		pCfB8622	NatMX	ADE2	YPDGN	
9	Positive control		pTAJAK-71	NatMX	Episomal	YPDGN	
10	Negative control					YPDGN	
11	Extra *crtI*	pCfB8786 (*IntD1::XdcrtI)*	pCfB6631	NatMX	Int_D1	YPDN	ST8889
12	YlHMG1	pCfB8787 (*IntD1::YlHMG1*)	pCfB6631	NatMX	Int_D1	YPDN	
13	Extra *XdcrtI* + YlHMG1	pCfB8788 (*IntD1::XdcrtI-YlHMG1)*	pCfB6631	NatMX	Int_D1	YPDN	
14	EasyClone integration minus control		pCfB6631	NatMX	Int_D1	YPDN	
15	Positive control		pCfB3405	NatMX	Int_D1	YPDN	
16	Negative control					YPDN	

### Wednesday (Day 3)

Today, students will purify PCR confirmed EasyClone plasmids and prepare them for transformation into yeast. Students will also prepare plates for transformation and inoculate agar plates with the parental yeast strains.

1. Prepare EasyClone plasmids for yeast transformation by first purifying the plasmid DNA using a NucleoSpin Plasmid kit (or similar) according to the manufacturer's instructions.
  - Prepare plasmids for genomic integration by digesting with FastDigest NotI restriction enzyme:
    - Add 1 U per μg of plasmid (for some plasmids up to 4 μg of integration fragment is required so we suggest digesting as much DNA as possible).
    - Add an appropriate amount of FastDigest buffer.
    - Incubate at 37°C for 1 h.
    - Heat inactivate the restriction enzyme at 65°C for 20 min.
    - Measure the concentration using NanoDrop (or similar).
2. Prepare agar plates for transformation.
  - For the *S. cerevisiae* transformation (ST7574) prepare YPD agar plates with 20 g L^−1^ agar and 200 mg L^−1^ G418 (YPDG) and YPD agar plates with 20 g L^−1^ agar, 200 mg L^−1^ G418 and 100 mg L^−1^ Nourseothricin (YPDGN).
  - For the *Y. lipolytica* transformation (ST8889) prepare YPD agar plates with 20 g L^−1^ agar and 250 mg L^−1^ Nourseothricin (YPDN).
3. Prepare yeast parental strains for transformation by restreaking ST7574 onto YPDG and ST8889 onto YPD agar plates. Incubate plates at 30°C for 2 days.

Alternate protocol

1. If time and resources allow, you can additionally confirm correct plasmid assembly by DNA sequencing the plasmids using the same primers listed in Table 4. Follow the protocol given by your chosen sequencing provider. Map the sequencing reads to the plasmid maps provided in the supplementary materials (Supporting Information).

### Thursday (Day 4)

Today, students can make sure everything is ready for transformation of the DNA constructs into yeast. The day can also be used to repeat any experiments that may have failed in the previous days.

### Friday (Day 5)

Today, students will transform the DNA constructs they prepared during the week into yeast and incubate over the weekend to allow single colonies to form. Expression cassettes will be introduced according to the scheme outlined in Table 6.

1. *S. cerevisiae* transformation
  - Transfer cells from the YPDG agar plate to 5 mL of fresh YPDG liquid media (YP media + 20 g L^−1^ glucose + 200 mg L^−1^ G418).
  - Measure OD600 of the 5 mL culture and use to inoculate 50 mL of fresh YPDG media to a starting OD600 of 0.4. Incubate at 30°C with shaking at 200 RPM until an OD600 of 1.6 is reached (∼3–4 h).
  - Harvest cells by centrifugation at 5000 g for 5 min and wash twice in 20 mL sterile H_2_O. Resuspend cells to a final volume of ∼1 mL and transfer to a sterile 1.5 mL Eppendorf tube.
  - Gently spin down cells in a microcentrifuge for 10 s at 2500 x g and resuspend in 450 μL sterile H_2_O. Keep cells on ice until use.
  - Add DNA parts for transformation into *S. cerevisiae* strain ST7574 as outlined in Table 6 into sterile 1.5 mL Eppendorf tubes:
    - Add 1000 ng of integration fragment DNA.
    - Add 200 ng of plasmid DNA.
    - Add H_2_O to a final volume of 24 μL.
  - Add 50 μL of cells to each tube containing DNA.
  - Add the following transformation reagents to each tube.
  - Gently vortex and then incubate at 30°C for 30 min.
  - Gently mix and then incubate at 42°C for 40 min.
  - Gently spin down cells in a microcentrifuge for 10 s at 2500 x g and resuspend in 500 μL YPDG. Incubate at 30°C for at least 2 h with gentle shaking.
  - Gently spin down cells in a microcentrifuge for 10 s at 2500 x g, remove 450 μL of media, resuspend cells in the remaining 50 μL and plate on YPD + G418 + Nourseothricin agar plates (YPDGN).
  - Incubate at 30°C for ∼3 days until single colonies are visible.
2. *Y. lipolytica* transformation
  - Transfer cells from the YPD agar plate into 1 mL of sterile H_2_O.
  - Harvest cells by centrifugation for 5 min at 3000 g, wash twice in 1 mL sterile H_2_O.
  - Resuspend the cells in 1 mL sterile H_2_O and measure OD600.
  - For one transformation reaction, an equivalent OD600 of 9.2 is required. For example, if the OD600 of your 1000 µL cell suspension is 40, 230 µL of the cell suspension equals an equivalent OD600 of 9.2 ((1000 µL/40)*9.2 = 230 µL).
  - Transfer the required volume for each transformation to a sterile Eppendorf tube, centrifuge for 5 min at 3000 g at room temperature and remove the supernatant.
  - Add 1000 ng of linearized integration vector DNA and 500 ng of plasmid DNA to the cell pellet according to the scheme outlined in Table 6.
  - Gently resuspend the cell pellet in the following transformation mix.

**Table:** 

Reagent	Volume (μL)
PEG 3500 50% (w/v)	240
LiAc 1 M	36
Boiled ssDNA (10 mg mL^−1^)	10

**Table:** 

Reagent	Volume (μL)
PEG 3500 50% (w/v)	87.5
LiAc 2 M	5
Boiled ssDNA (10 mg mL^−1^)	2.5

  h. Incubate the cells at 39°C for 60 min.
  i. Spin down the cells for 5 min at 3000 g at room temperature and resuspend in 500 µL YPD. Incubate at 30°C for at least 2 h with gentle shaking.
  j. Spin down the cells for 5 min at 3000 g at room temperature, remove 450 µL of YPD media and resuspend the cell pellet in the remaining 50 µL. Plate cells on YPDN.
  k. Incubate at 30°C for ∼3 days until single colonies are visible.

### Monday (Week 2, Day 8)

**Figure 6.**
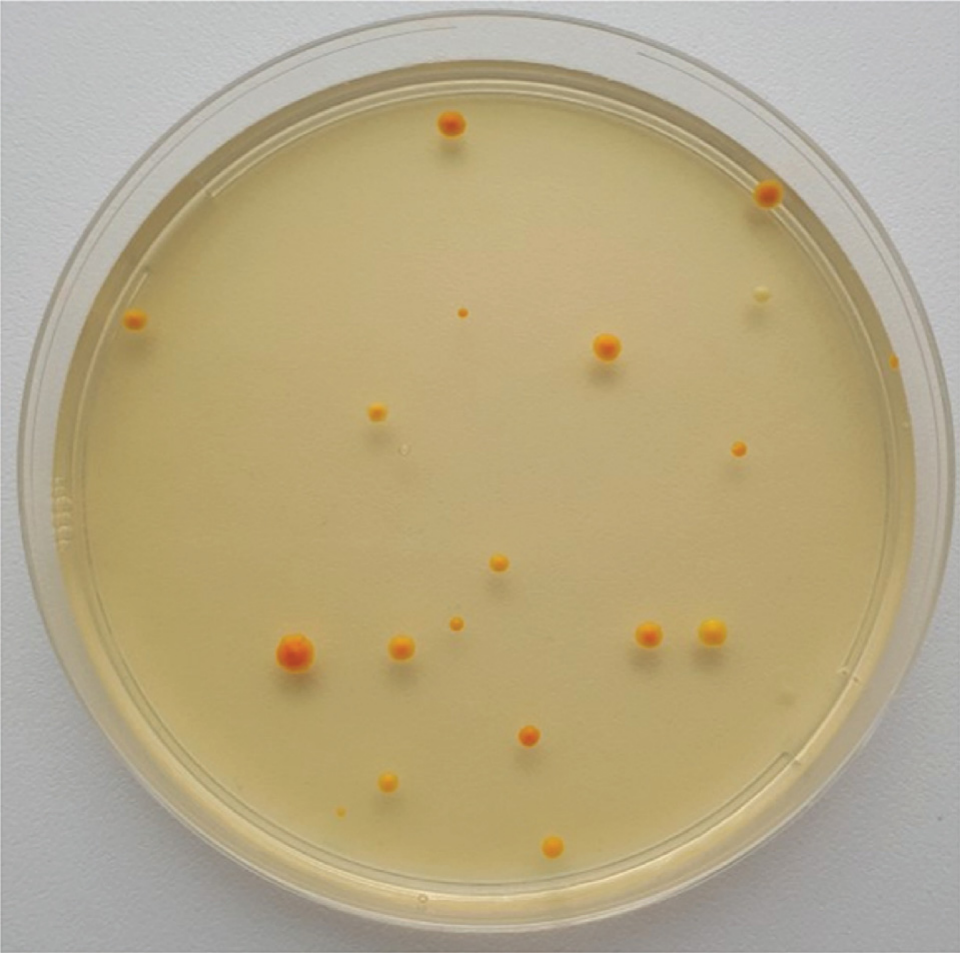
A successful transformation plate showing yeast cells that have integrated the β-carotene pathway.

Over the weekend, single colonies should appear on the transformation plates. For the introduction of genes involved in β-carotene synthesis, successfully integrated colonies should appear shades of yellow, orange or red due to the buildup of β-carotene and its intermediates (Verwaal *et al*. 2007); an example of a transformation plate is shown in Fig. 6. For the *ADE2* knockout transformation, successful clones should appear red due to the buildup of a red pigment from the adenine biosynthesis pathway (Ugolini and Bruschi 1996). Today, students will do colony PCR (cPCR) to confirm the genotype of their transformants. Because some transformations introduced DNA at two or three different locations in the genome, multiple PCR reactions will need to be performed for each clone tested. Once students have confirmed which clones have the correct genome modifications, they can inoculate preculture media with the correct clones to start a production assay the following day to determine how the different engineering strategies impact β-carotene production. Diagnostic primers used for genotyping bind outside the genomic integration site (Out Fwd and Out Rev) and bind in the integration fragment (In Rev).

1. Set up cPCR reactions.
  - Prepare cPCR reactions according to the outline in Table 7.
2. Prepare cells for cPCR reaction.
  - Select several colonies from the transformation plate (in general the most orange colonies correlate to higher β-carotene production). Circle and number each colony tested.
  - Take a small amount of cells and transfer to a PCR tube containing 6 µL of H_2_O. Boil cells in a PCR machine for 10 min and then spin down tubes to collect cell pellet.
  - Transfer 2 µL of supernatant to each PCR reaction as outlined below.

**Table:** 

T	Volume (μL)
OneTaq 2x MM	10
Primer 1	1
Primer 2	1
Primer 3	1
H_2_O	5
DNA	2

  d. Run the PCR in the following program.

**Table:** 

t	Temp (°C)	Time (s)
	95	300
30x	94	30
	52	30
	68	180
	68	300

  e. Analyze the samples on 1% agarose gel (for corresponding PCR product size, see Table 7). Include a GeneRuler 1 Kb DNA ladder. A gel image showing a successful cPCR and correct integration of each cassette is given in Fig. 7.
3. Prepare correct clones for β-carotene production assay.
  - Inoculate 1 mL YPD preculture media with at least one correct clone from each transformation in a 10 mL preculture tube (Greiner) and incubate overnight 30°C with shaking at 200 RPM.

**Figure 7.**
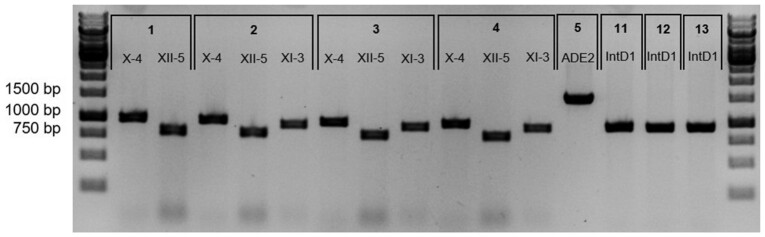
Gel image of a successful colony PCR amplification and correct integration of each cassette. Numbers correspond to those presented in Table 7 with each integration site given below. Negative image given for clarity.

**Table 7.** cPCR layout to genotype transformants.

			Diagnostic primers			Expected size		
#	Introduction	Integration fragments	Out Fwd	Out Rev	In Rev	Correct	Incorrect	Parental strain
1	Beta-carotene pathway	pCfB8379 (*X-4::XdcrtI-XdcrtYB*)	PR-905	PR-906	PR-2221	983	1394	ST7574
		pCfB8380 (*XII-5::XdcrtE*)	PR-899	PR-900	PR-2221	811	1365	
2	Beta-carotene pathway + Extra *XdcrtI*	pCfB8379 (*X-4::XdcrtI-XdcrtYB*)	PR-905	PR-906	PR-2221	983	1394	
		pCfB8380 (*XII-5::XdcrtE*)	PR-899	PR-900	PR-2221	811	1365	
		pCfB8382 (*XI-3::XdcrtI*)	PR-911	PR-912	PR-2221	927	1450	
3	Beta-carotene pathway + tHMG1	pCfB8379 (*X-4::XdcrtI-XdcrtYB*)	PR-905	PR-906	PR-2221	983	1394	
		pCfB8380 (*XII-5::XdcrtE*)	PR-899	PR-900	PR-2221	811	1365	
		pCfB8383 (*XI-3::tHMG1*)	PR-911	PR-912	PR-2221	927	1450	
4	Beta-carotene pathway + extra *XdcrtI* + tHMG1	pCfB8379 (*X-4::XdcrtI-XdcrtYB*)	PR-905	PR-906	PR-2221	983	1394	
		pCfB8380 (*XII-5::XdcrtE*)	PR-899	PR-900	PR-2221	811	1365	
		pCfB8381 (*XI-3::XdcrtI-tHMG1*)	PR-911	PR-912	PR-2221	927	1450	
5	ADE2 knockout	PR-23173 (ADE2 dsDNA repair fragment)	PR-7085	PR-7086		1512	3228	
11	Extra *XdcrtI*	pCfB8786 (*IntD1::XdcrtI)*	PR-14832	PR-14564	PR-8859	927	1145	ST8889
12	YlHMG1	pCfB8787 (*IntD1::YlHMG1*)	PR-14832	PR-14564	PR-8859	927	1145	
13	Extra *XdcrtI* + YlHMG1	pCfB8788 (*IntD1::XdcrtI-YlHMG1)*	PR-14832	PR-14564	PR-8859	927	1145	

Alternate protocol

1. It can sometimes be difficult to extract DNA of high enough quality for successful PCR by simply boiling cells in water. If time allows (e.g. if you have more than two weeks to run the protocol), we suggest preparing genomic DNA for PCR by a more robust method. In particular, we recommend the LiAc SDS method presented below as it is cheap, fast, reliably produces high quality DNA and is amenable to high-throughput in 96-well plate format. Extract genomic DNA as follows.
  - Inoculate 400 μL of YPD in a 96-deep-well plate with single colonies. Incubate overnight at 30°C with shaking at 200 RPM.
  - Transfer 100 μL of cell culture to a fresh 96-deep-well plate and set aside to use later as a preculture for the β-carotene production assay.
  - Spin down the remaining 400 μL of cell culture and resuspend in 100 μL of 20 mM LiAc, 1% SDS solution. Incubate at 70°C for 15 min with vortexing every 5 min to break open the cell wall.
  - Add 300 μL of 100% ethanol to each well and vortex well.
  - Spin down then wash cells in 500 μL of 70% ethanol.
  - Remove supernatant and allow residual ethanol to evaporate by incubating at 37°C until ethanol smell has disappeared (∼15 min).
  - Resuspend cells in 50 μL H_2_O, spin down and transfer supernatant (containing gDNA) to a fresh PCR plate to use later as PCR template.
2. Run a PCR to confirm correct genome integration described above.
3. Since both parental strains are prototrophic both the preculture and subsequent production assay can be performed in synthetic media (SM). While the strains will likely not grow as fast, the clear media will allow the carotenoid pigments to be more visible.

### Tuesday (Day 9)

By today, you should have yeast precultures of various colors of yellow, orange and red. Today, you will start a production assay to measure how much β-carotene is produced by each strain and use these results to assess the relative success of each genomic integration.

1. Start a β-carotene production assay with precultures from each successful transformation.
  - Inoculate with strains from preculture in duplicate in 2.5 mL YPD media with 8% glucose to a starting OD600 of ∼0.1 in a 24-deep-well cultivation plate.
  - Incubate for ∼48 h at 30°C with shaking at 200 RPM.

Alternate protocol

1. If time allows, incubating cells for 72 h may improve titers.
2. If 24-deep-well plates are not available, you can cultivate the strains in e.g. shake flask or any other system with a minimum volume of 2.5 mL.

### Wednesday (Day 10)

No experimental work is scheduled on these days. We suggest spending the time showing students the *in silico* design of gRNA plasmids using online tools such as CRISPy (http://staff.biosustain.dtu.dk/laeb/crispy_cenpk/) (Jakočiūnas *et al*. 2015). This protocol only scratches the surface of possible engineering targets to boost flux toward β-carotene; a further suggestion would be to ask students to perform a literature search to identify additional metabolic engineering strategies and make a workflow for creating these additionally boosted strains.

### Thursday (Day 11)

Today, students will prepare samples to measure β-carotene production. Unlike many other value-added molecules produced by engineered yeasts, β-carotene is not exported in significant amounts from the cell and thus accumulates intracellularly. In order to quantify β-carotene production, students will first need to lyse the yeast cells to extract the intracellular product. A further complication is that β-carotene is highly insoluble in water, so extraction is performed using ethyl acetate. Note that ethyl acetate is highly volatile and flammable so care must be taken when handling. Perform all steps in a fume hood using nitrile gloves and eye protection. Read the SDS before use.

The extraction protocol used is according to Kildegaard *et al*. (2017) and is described below.

1. Measure cell dry weight.
  - Transfer 1 mL of the cultivation broth into a preweighed 2 mL microtube.
  - Centrifuge at 10 000 g for 5 min. Remove the supernatant and place the tubes. containing the biomass pellets in the incubator at 60°C for 24 h. After 24 h, weigh the tubes on an analytical scale.
2. Extract β-carotene from engineered yeast strains.
  - Transfer 500 µL of culture into a prelabeled 2 mL microtube (Sarstedt) for β-carotene extraction.
  - Centrifuge the tubes at 10,000 g for 10 min and remove the supernatant.
  - Add 0.5 mL of 0.5–0.75 mm glass beads to each tube followed by the addition of 0.5 mL of ethyl acetate supplemented with 0.01% 3,5-di-tert-4- butylhydroxytoluene (BHT). BHT is added to prevent carotenoid oxidation.
  - Disrupt the cells using the Precellys R 24 homogenizer (Bertin Corp.) in four cycles of 5500 RPM for 20 s. Cool the tubes by placing on ice for 1 min in between each lysis cycle. After disruption, centrifuge cells for 10 min at 10 000 g.
  - For quantification of β-carotene by HPLC, transfer 100 µL of the solvent fraction to HPLC vials.
  - Evaporate the 100 µL of ethyl acetate extract in a rotatory evaporator (SpeedVac) for at least 45 min. Redissolve the dry extracts in 1 mL of 99% ethanol + 0.01% BHT.
3. Prepare β-carotene standards in 100% ethanol with 0.01% BHT.
  - Prepare an initial 30 mg L^−1^ stock solution in ∼5 mL.
  - Prepare dilution standards according to the scheme below.

**Table:** 

Final conc. (mg/L)	Stock vol (μL)	Ethanol + BHT vol (μL)
21	700	300
18	600	400
15	500	500
12	400	600
9	300	700
6	200	800
3	100	900
1.5	50	950

4. Run samples on HPLC.
  a. The extracts can now be analyzed by HPLC (ThermoFisher Scientific). The machine is equipped with a Discovery HS F5 150 mm x 2.1 mm column (particle size 3 mm). For β-carotene analysis, the column oven temperature is set to 30°C. The flow rate is set to 0.7 mL min^−1^ with an initial solvent composition of 10 mM ammonium formate 157 (pH = 3, adjusted with formic acid) (solvent A) and acetonitrile (solvent B) (3:1) until minute 2.0. Solvent composition is then changed at minute 4.0 following a linear gradient until %A = 10.0 and %B = 90.0. The solvent composition is kept until 10.5 min when the solvent returns to initial conditions and the column is re-equilibrated until 13.5 min. The injection volume is 10 µL (Kildegaard *et al*. 2017).
  b. The peaks obtained from the sample analysis can now be identified by comparison to prepared standards. Check the peaks for each sample to confirm the right integration of the peak areas. If necessary, do the integration manually. β-carotene is detected at a retention time of ∼7.6 min, by measuring absorbance at 450 nm.

Alternate protocol

If access to an HPLC machine is infeasible, quantification can also be achieved using a plate-reader or cuvette based spectrophotometer.

1. Follow the protocol as described above, after redissolving the dry extracts in 1 mL of 99% ethanol + 0.01% BHT and preparing the β-carotene standards, measure the absorbance at 450 nm using a spectrophotometer. Use the β-carotene standards to determine the linear range of your machine and dilute samples as needed to be within this range. Use a 1 mL solution of 99% ethanol + 0.01% BHT as a blank.

### Friday (Day 12)

Today, students will analyze the HPLC results and determine the β-carotene yield of the different strains constructed. With these results, students should then be able to make conclusions about the relative success of each metabolic engineering strategy and come up with hypotheses to explain the results.

1. Measure the cell dry weight as outlined above.
2. Determine the amount of β-carotene produced per gram of cell dry weight.

## EXPECTED RESULTS

**Figure 8.**
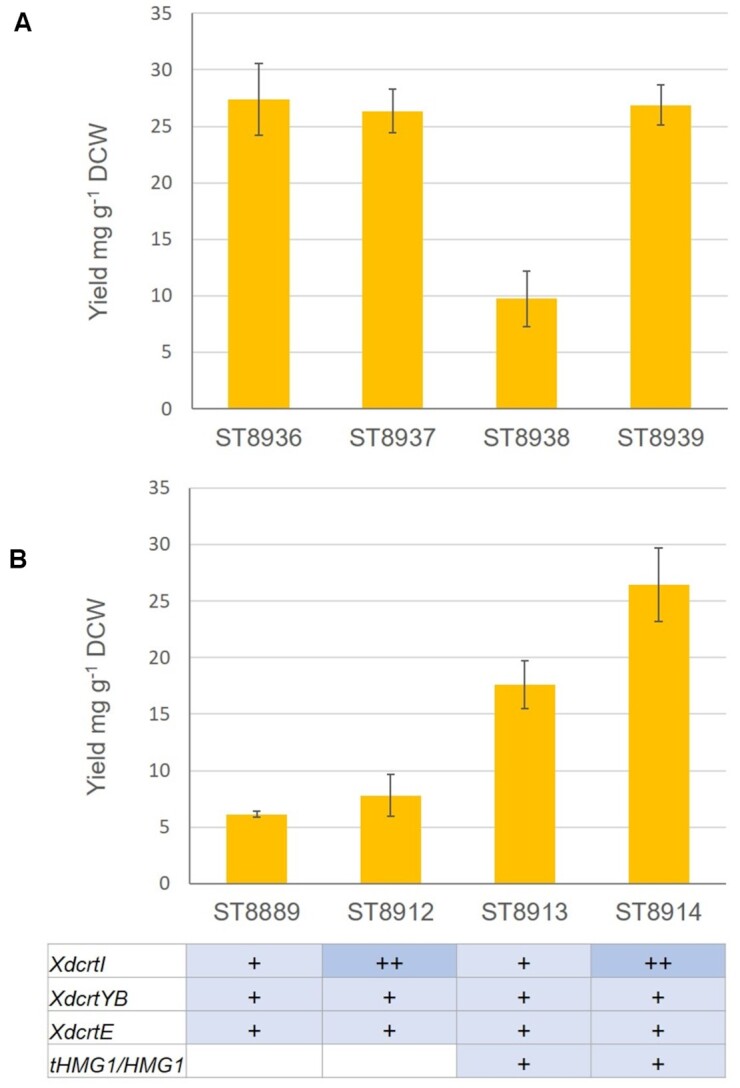
β-carotene yields from engineered *S. cerevisiae***(A)** and *Y. lipolytica***(B)** strains. Data presented as averages and standard deviations from quadruplicate experiments.

Figure 8 shows the results obtained when the authors ran the experiment. While the results for *Y. lipolytica* strains matched our expectations with an increase in *XdCrtI* and Hmg1 activity correlating with higher β-carotene yields, surprisingly the same trend was not observed for *S. cerevisiae* strains. While Verwaal *et al*.(2007) reported an increase in β-carotene levels upon overexpression of tHMG1, Ronda *et al*. (2015) reported a decrease in β-carotene with tHMG1 overexpression similar to the results presented here. Since we only measure β-carotene in this experiment, the observed discrepancies could be due to an increase in the production of phytoene and lycopene which could not be further converted to β-carotene.

## Supplementary Material

foz062_Supplement_File
