# Supplementary material for: A teaching protocol demonstrating the use of EasyClone and CRISPR/Cas9 for metabolic engineering of Saccharomyces cerevisiae and Yarrowia lipolytica
Source: FEMS Yeast Res. 2019 Sep 26;20(2):foz062. doi: 10.1093/femsyr/foz062 (PMC8260333; doi:10.1093/femsyr/foz062)
Supplement: foz062_Supplement_File [file foz062_supplement_file.docx]

**Supplementary materials**

**Supplementary table 1.** All strains used in this publication.

| Name | Relevent genotype | Origin | Euroscarf ID |
| --- | --- | --- | --- |
|  |  |  |  |
| *S. cerevisiae* | | |  |
|  |  |  |  |
| CEN.PK113-7D | MATa *URA3 HIS3 LEU2 TRP1 MAL2-8c SUC2* | (Entian & Kötter, 2007) | NA |
| ST7574 | MATa *URA3 HIS3 LEU2 TRP1 MAL2-8c SUC2* + pCfB2312 (2μm *cas9* KanMX) | This study | Y41390 |
| ST8936 | MATa *URA3 HIS3 LEU2 TRP1 MAL2-8c SUC2 X-4::XdCrtI-XdCrtYB XII-5::XdCrtE* + pCfB2312 (2μm *cas9* KanMX) | This study | Y41391 |
| ST8937 | MATa *URA3 HIS3 LEU2 TRP1 MAL2-8c SUC2 X-4::XdCrtI-XdCrtYB XII-5::XdCrtE XI-3::XdCrtI* + pCfB2312 (2μm *cas9* KanMX) | This study | Y41392 |
| ST8938 | MATa *URA3 HIS3 LEU2 TRP1 MAL2-8c SUC2 X-4::XdCrtI-XdCrtYB XII-5::XdCrtE XI-3::tHMG1* + pCfB2312 (2μm *cas9* KanMX) | This study | Y41393 |
| ST8939 | MATa *URA3 HIS3 LEU2 TRP1 MAL2-8c SUC2 X-4::XdCrtI-XdCrtYB XII-5::XdCrtE XI-3::XdCrtI-tHMG1* + pCfB2312 (2μm *cas9* KanMX) | This study | Y41394 |
|  |  |  |  |
| *Y. lipolytica* | | |  |
|  |  |  |  |
| W29 (ATCC 20460) | MATa | (Pomraning & Baker, 2015) | NA |
| ST6512 | MATa *ku70∆::cas9-DsdA* | This study | NA |
| ST8894 | MATa *ku70∆::cas9-DsdA IntC2::XdCrtI-XdCrtYB* | This study | Y41395 |
| ST8889 | MATa *ku70∆::cas9-DsdA IntC2::XdCrtI-XdCrtYB IntE1::XdCrtE* | This study | Y41396 |
| ST8912 | MATa *ku70∆::cas9-DsdA IntC2::XdCrtI-XdCrtYB IntE1::XdCrtE IntD1::XdCrtI* | This study | Y41397 |
| ST8913 | MATa *ku70∆::cas9-DsdA IntC2::XdCrtI-XdCrtYB IntE1::XdCrtE IntD1::HMG1* | This study | Y41398 |
| ST8914 | MATa *ku70∆::cas9-DsdA IntC2::XdCrtI-XdCrtYB IntE1::XdCrtE IntD1::XdCrtI-HMG1* | This study | Y41399 |

**Supplementary table 2.** All plasmids used in this publication.

| Name | Relevant characteristics | Origin | Addgene ID |
| --- | --- | --- | --- |
|  |  |  |  |
| Templates for PCR amplification | | |  |
|  |  |  |  |
| p1977 | pUC19 + pTDH3-pTEF1 | (Jessop-Fabre et al., 2016) | 78230 |
| pCfB8739 | pUC19 + *XdCrtYB* | This study | 126905 |
| pCfB8740 | pUC19 + *XdCrtI* | This study | 126906 |
| pCfB8741 | pUC19 + *XdCrtE* | This study | 126907 |
| pCfB8742 | pUC19 + pGPD1-pFBA1 | This study | 126908 |
| pCfB8818 | pUC19 + YlHMG1 | This study | 126909 |
| pCfB8792 | pUC19 + pSNR52-ADE2 gRNA-tSUP4 | This study | 126910 |
|  |  |  |  |
| gRNA plasmids for targeting genomic integration sites by CRISPR-Cas9 | | | |
|  |  |  |  |
| pCfB5191 | 2μm ori NatMX pSNR52-X-4 gRNA-tSUP4 pSNR52-XII-5 gRNA-tSUP4 | This study | 126911 |
| pCfB8622 | 2μm ori NatMX pSNR52-ADE2 gRNA-tSUP4 | This study | 126912 |
| pCfB3052 | 2μm ori NatMX pSNR52-X-4 gRNA-tSUP4 pSNR52-XII-5 gRNA-tSUP4 pSNR52-XI-3 gRNA-tSUP4 | (Jessop-Fabre et al., 2016) | 73294 |
| pCfB6631 | 2μm ori NatMX pSNR52-IntD1 gRNA-tSUP4 | (Holkenbrink et al., 2017) | 106161 |
| pCfB6627 | 2μm ori NatMX pSNR52-IntC2 gRNA-tSUP4 | (Holkenbrink et al., 2017) | 106159 |
| pCfB6633 | 2μm ori NatMX pSNR52-IntE1 gRNA-tSUP4 | (Holkenbrink et al., 2017) | 106162 |
|  |  |  |  |
| Episomal yeast expression plasmids | | |  |
|  |  |  |  |
| pCfB2312 | 2μm ori c*as9* KanMX | (Jessop-Fabre et al., 2016) | 78231 |
| pTAJAK-71 | 2μm ori NatMX | (Jessop-Fabre et al., 2016) | 78232 |
| pCfB3405 | 2μm ori NatMX | (Holkenbrink et al., 2017) | 106166 |
|  |  |  |  |
| Backbone plasmids for EasyClone-MarkerFree plasmid assembly | | | |
|  |  |  |  |
| pCfB3035 | pX-4-USER | (Jessop-Fabre et al., 2016) | 73273 |
| pCfB2909 | pXII-5-USER | (Jessop-Fabre et al., 2016) | 73281 |
| pCfB2904 | pXI-3-USER | (Jessop-Fabre et al., 2016) | 73276 |
| pCfB6677 | pIntE_1-USER | (Holkenbrink et al., 2017) | 106155 |
| pCfB6682 | pIntC_2-USER | (Holkenbrink et al., 2017) | 106152 |
| pCfB6684 | pIntD_1-USER | (Holkenbrink et al., 2017) | 106154 |
| pTAJAK-71 | 2μm ori NatMX | (Jessop-Fabre et al., 2016) | 78232 |
|  |  |  |  |
| Plasmids for integration into yeast genome | | | |
|  |  |  |  |
| *S. cerevisiae* | | |  |
| pCfB8379 | *X-4::XdCrtI<-pTDH3-pTEF1->XdCrtYB* | This study | 126913 |
| pCfB8380 | *XII-5:: pPGK1->XdCrtE* | This study | 126914 |
| pCfB8381 | *XI-3::XdCrtI<-pTDH3-pTEF1->tHMG1* | This study | 126915 |
| pCfB8382 | *XI-3::XdCrtI<-pTDH3* | This study | 126916 |
| pCfB8383 | *XI-3::pTEF1->tHMG1* | This study | 126917 |
| *Y. lipolytica* | | |  |
| pCfB6364 | *ku70∆::cas9-DsdA* | (Holkenbrink et al., 2017) | 106151 |
| pCfB8748 | *IntE1::pFBA1->CrtE* | This study | 126918 |
| pCfB8644 | *IntC2::XdCrtI<-pGPD-pFBA1->XdCrtYB* | This study | 126919 |
| pCfB8786 | *IntD1::XdCrtI<-pGPD* | This study | 126920 |
| pCfB8787 | *IntD1::pFBA1->HMG1* | This study | 126921 |
| pCfB8788 | *IntD1::XdCrtI<-pGPD-pFBA1->HMG1* | This study | 126922 |
|  |  |  |  |

**Supplementary table 3.** All primers used in this publication

| Name | Sequence (5'→3') | Purpose |
| --- | --- | --- |
|  |  |  |
| Primers for biobrick amplification | | |
|  |  |  |
| PR-7039 | ATCTGTCAUATGACGGCTCTCGCATATTA | Fwd primer for amplification of BB01567 (XdCrtYB) |
| PR-7040 | CACGCGAUTTACTGCCCTTCCCATCCGC | Rev primer for amplification of BB01567 (XdCrtYB) |
| PR-7041 | AGTGCAGGUATGGGAAAAGAACAAGATCAGG | Fwd primer for amplification of BB01568 (XdCrtI) |
| PR-7042 | CGTGCGAUTCAGAAAGCAAGAACACCAACG | Rev primer for amplification of BB01568 (XdCrtI) |
| PR-7043 | ATCTGTCAUATGGATTACGCGAACATCCTC | Fwd primer for amplification of BB01569 (XdCrtE) |
| PR-7044 | CACGCGAUTCACAGAGGGATATCGGCTAG | Rev primer for amplification of BB01569 (XdCrtE) |
| PR-22921 | ATCTGTCAUAAAACAATGACTGCAGACCAATTGGTG | Fwd primer for amplification of BB3287(tHMG1) |
| PR-22922 | CACGCGAUTCAGGATTTAATGCAGGTGACG | Rev primer for amplification of BB3287(tHMG1) |
| PR-1853 | ACCTGCACUTTTGTTTGTTTATGTGTGTTTATTC | Fwd primer for amplification of BB0464 (<-pTDH3-pTEF1->) |
| PR-22409 | ATGACAGAUTTGTAATTAAAACTTAGATTAGATTG | Rev primer for amplification of BB0464 (<-pTDH3-pTEF1->) |
| PR-22406 | CGTGCGAUGGAAGTACCTTCAAAGAATGG | Fed primer for amplification of BB0009 (pPGK1->) |
| PR-22407 | ATGACAGAUTTGTTTTATATTTGTTGTAAAAAGTAG | Rev primer for amplification of BB0009 (pPGK1->) |
| PR-1852 | CACGCGAUATAAAAAACACGCTTTTTCAG | Fwd primer for amplification of BB0410 (<-pTDH3) |
| PR-1853 | ACCTGCACUTTTGTTTGTTTATGTGTGTTTATTC | Rev primer for amplification of BB0410 (<-pTDH3) |
| PR-23752 | ATCTGTCAUGCCACA ATGCTACAAGCAGCTATTGG | Fwd primer for amplification of BB3787 (YlHMG1->) |
| PR-23753 | CACGCGAUCTATGACCGTATGCAAATATTCG | Rev primer for amplification of BB3787 (YlHMG1->) |
| PR-13337 | CACGCGAUGACGCAGTAGGATGTCCTG | Fwd primer for amplification of BB1244 (<-pGPD) |
| PR-13338 | ACCTGCACUGTTGATGTGTGTTTAATTC | Rev primer for amplification of BB1244 (<-pGPD) |
| PR-15523 | CGTGCGAUAACAGTGTACGCAGTACTATAGAGG | Fwd primer for amplification of BB1559 (pFBA1->) |
| PR-15524 | ATGACAGAUTGTGTGATGTGTAGTTTAGATTTCG | Rev primer for amplification of BB1559 (pFBA1->) |
| PR-10525 | CGTGCGAUAGGGAACAAAAGCTGGAGCT | Fwd primer for amplification of BB3713(ADE2 gRNA knockout cassette) |
| PR-10529 | CACGCGAUTAACTAATTACATGACTCGA | Rev primer for amplification of BB3713(ADE2 gRNA knockout cassette) |
|  |  |  |
| Primers for verification of correct EasyClone plasmid assembly | | |
|  |  |  |
| PR-22955 | GACGGTAGGTATTGATTGTAATTCTG | pTDH3 Fwd diagnostic PCR primer |
| PR-339 | GCTCATTAGAAAGAAAGCATAGC | pTEF1 Fwd diagnostic PCR primer |
| PR-340 | TACAGATCATCAAGGAAGTAATTATC | pPGK1 Fwd diagnostic PCR primer |
| PR-224 | GAAATTCGCTTATTTAGAAGTGTC | tADH1 Rev diagnostic PCR primer |
| PR-225 | CTCCTTCCTTTTCGGTTAGAG | tCYC1 Rev diagnostic PCR primer |
| PR-23875 | ACTGTTGGGAAGGGCGATC | gRNA cassette Fwd diagnostic PCR primer |
| PR-23876 | AGCGCCCAATACGCAAAC | gRNA cassette Rev diagnostic PCR primer |
| PR-14441 | GTCTGCATCGCCGGCTC | pGPD1 Fwd diagnostic PCR primer |
| PR-15587 | CTAGGGTATATATAAACAGTGGCTC | pFBA1 Fwd diagnostic PCR primer |
| PR-14617 | TATCCCTGTGTTGAATC | tPEX20 Rev diagnostic PCR primer |
| PR-14619 | TATCGACCCAGTTAGC | tLIP2 Rev diagnostic PCR primer |
|  |  |  |
| Primers for genotyping correct genomic integration of expression cassettes | | |
|  |  |  |
| PR-2221 | GTTGACACTTCTAAATAAGCGAATTTC | Universal Rev primer binding in *S. cerevisiae* integration cassettes |
| PR-905 | CTCACAAAGGGACGAATCCT | Fwd primer for diagnostic PCR of EasyClone plasmid integration at X-4 site |
| PR-906 | GACGGTACGTTGACCAGAG | Rev primer for diagnostic PCR of EasyClone plasmid integration at X-4 site |
| PR-911 | GTGCTTGATTTGCGTCATTC | Fwd primer for diagnostic PCR of EasyClone plasmid integration at XI-3 site |
| PR-912 | CACATTGAGCGAATGAAACG | Rev primer for diagnostic PCR of EasyClone plasmid integration at XI-3 site |
| PR-899 | CCACCGAAGTTGATTTGCTT | Fwd primer for diagnostic PCR of EasyClone plasmid integration at XII-5 site |
| PR-900 | GTGGGAGTAAGGGATCCTGT | Rev primer for diagnostic PCR of EasyClone plasmid integration at XII-5 site |
| PR-7085 | CCTCTATCTTCAAACGAATC | Fwd primer for diagnostic PCR to confirm ADE2 deletion |
| PR-7086 | TAACGGAACAAGATCTCAAC | Rev primer for diagnostic PCR to confirm ADE2 deletion |
| PR-8859 | AAGTGTGGATGGGGAAGTGAG | Universal Rev primer binding in *Y. lipolytica* integration cassettes |
| PR-14832 | ACTGGTGGCTACAAATGAAG | Fwd primer for diagnostic PCR of EasyClone plasmid integration at Int_D1 site |
| PR-14564 | CACGCGAUCCGGCCAGGGGTAAGAG | Rev primer for diagnostic PCR of EasyClone plasmid integration at Int_D1 site |
|  |  |  |
| gRNA repair dsDNA oligos | | |
|  |  |  |
| PR-23173 | TATAACAATCAAGAAAAACAAGAAAATCGGACAAAACAATCAAGTT ATATAAGTTTATTGATATACTTGTACAGCAAATAATTATAAAAT | dsDNA oligo repair fragment for ADE2 gene deletion |

Entian, K. D., & Kötter, P. (2007). 25 Yeast Genetic Strain and Plasmid Collections. *Methods in Microbiology*, *36*(06), 629–666. https://doi.org/10.1016/S0580-9517(06)36025-4

Holkenbrink, C., Dam, M. I., Kildegaard, K. R., Beder, J., Dahlin, J., Doménech Belda, D., & Borodina, I. (2017). EasyCloneYALI: CRISPR/Cas9-Based Synthetic Toolbox for Engineering of the Yeast Yarrowia lipolytica. *Biotechnology Journal*, *1700543*, 1–8. https://doi.org/10.1002/biot.201700543

Jessop-Fabre, M. M., Jakočiūnas, T., Stovicek, V., Dai, Z., Jensen, M. K., Keasling, J. D., & Borodina, I. (2016). EasyClone-MarkerFree: A vector toolkit for marker-less integration of genes into Saccharomyces cerevisiae via CRISPR-Cas9. *Biotechnology Journal*, *11*(8), 1110–1117. https://doi.org/10.1002/biot.201600147

Pomraning, K. R., & Baker, S. E. (2015). Draft Genome Sequence of the Dimorphic Yeast Yarrowia lipolytica Strain W29 . *Genome Announcements*, *3*(6), 10–11. https://doi.org/10.1128/genomea.01211-15
